# MicrobiomeAnalyst 2.0: comprehensive statistical, functional and integrative analysis of microbiome data

**DOI:** 10.1093/nar/gkad407

**Published:** 2023-05-11

**Authors:** Yao Lu, Guangyan Zhou, Jessica Ewald, Zhiqiang Pang, Tanisha Shiri, Jianguo Xia

**Affiliations:** Department of Microbiology and Immunology, McGill University, Quebec, Canada; Institute of Parasitology, McGill University, Quebec, Canada; Institute of Parasitology, McGill University, Quebec, Canada; Institute of Parasitology, McGill University, Quebec, Canada; Institute of Parasitology, McGill University, Quebec, Canada; Department of Microbiology and Immunology, McGill University, Quebec, Canada; Institute of Parasitology, McGill University, Quebec, Canada; Department of Animal Science, McGill University, Quebec, Canada

## Abstract

Microbiome studies have become routine in biomedical, agricultural and environmental sciences with diverse aims, including diversity profiling, functional characterization, and translational applications. The resulting complex, often multi-omics datasets demand powerful, yet user-friendly bioinformatics tools to reveal key patterns, important biomarkers, and potential activities. Here we introduce MicrobiomeAnalyst 2.0 to support comprehensive statistics, visualization, functional interpretation, and integrative analysis of data outputs commonly generated from microbiome studies. Compared to the previous version, MicrobiomeAnalyst 2.0 features three new modules: (i) a Raw Data Processing module for amplicon data processing and taxonomy annotation that connects directly with the Marker Data Profiling module for downstream statistical analysis; (ii) a Microbiome Metabolomics Profiling module to help dissect associations between community compositions and metabolic activities through joint analysis of paired microbiome and metabolomics datasets; and (iii) a Statistical Meta-Analysis module to help identify consistent signatures by integrating datasets across multiple studies. Other important improvements include added support for multi-factor differential analysis and interactive visualizations for popular graphical outputs, updated methods for functional prediction and correlation analysis, and expanded taxon set libraries based on the latest literature. These new features are demonstrated using a multi-omics dataset from a recent type 1 diabetes study. MicrobiomeAnalyst 2.0 is freely available at microbiomeanalyst.ca.

## Graphical Abstract

**Graphical Abstract:**
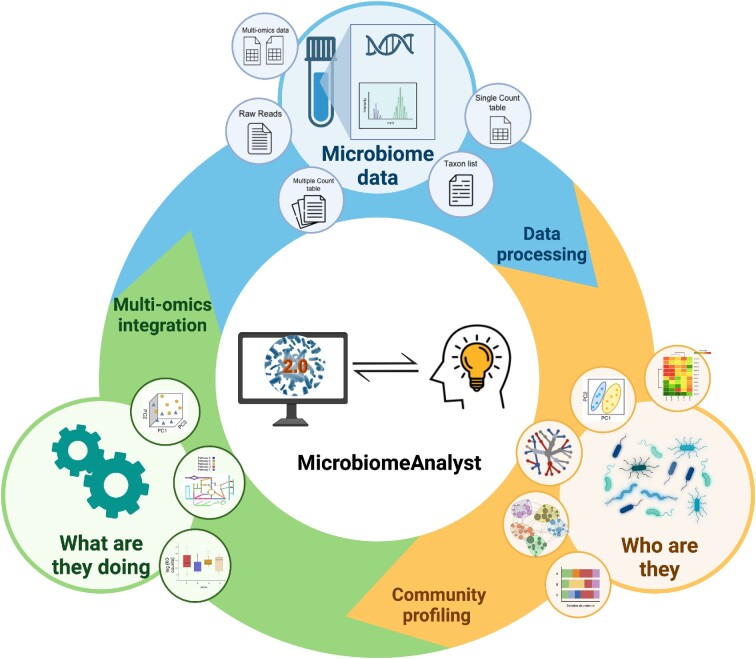

## INTRODUCTION

Over the past decade, microbiome studies have experienced tremendous growth across diverse disciplines with a clear trend towards leveraging multiple omics technologies for comprehensive characterization of the underlying communities (1,2). The microbiome is now considered a key player in human health and sustainable agriculture (3–6). Powerful bioinformatics pipelines and tools have been continuously developed and updated to help analyze increasingly complex datasets (7–10). Version 1.0 of MicrobiomeAnalyst was developed to provide a user-friendly web-based platform for bench researchers to perform comprehensive exploratory analysis on common abundance profiles and taxonomic signatures (11). Since its release in 2017, MicrobiomeAnalyst has been continuously updated based on user feedback, with a detailed analysis protocol published in 2020 (12). Based on Google Analytics, the MicrobiomeAnalyst public server has processed >125 000 jobs submitted from >30 000 users worldwide during the past 12 months.

Microbiome data analysis is conceptually similar to other omics data analysis workflows, consisting of three typical stages: raw data processing, statistical analysis, and functional interpretation. In practice, however, microbiome data shows much higher heterogeneity with particularly strong inter-individual and inter-population differences, causing statistical issues including zero inflation, compositionality and overdispersion (13–15). These characteristics have motivated the development of a wide array of analysis methods, resulting in a landscape challenging for researchers who are not experts in statistics or programming. The marker gene data analysis has seen a shift from the traditional operational taxonomy units (OTUs), which are clusters of reads based on similarity thresholds, towards high-resolution amplicon sequence variants (ASVs) identified based on their unique biological sequences (16). Using ASVs not only reduces the computational bottleneck associated with sequence clustering but also facilitates comparative analysis across different studies. In differential abundance analysis, several benchmark studies have shown inconsistencies among methods developed specifically for microbiome data, and that common RNAseq analysis methods are robust and perform well (17,18). Finally, there is a growing demand for easy-to-use yet flexible tools that can account for complex metadata as well as to support multi-omics integration for microbiome studies (19–21).

To keep up with the progress and the evolving data analysis needs arising from recent microbiome studies, we have made significant updates to the MicrobiomeAnalyst platform, including three new modules: (i) a raw data processing module for marker gene data that links directly to downstream statistical analysis; (ii) a microbiome metabolomics module for analysis of paired microbiome and metabolomics data, and (iii) a statistical meta-analysis module for multiple marker gene datasets. We have also made significant updates to the previous modules including support for complex metadata (metadata editor, continuous metadata, and multi-factor comparison analysis), enhanced statistical approaches (functional prediction and correlation network analysis), new interactive visualizations (stacked bar plot, heatmaps and a KEGG metabolic network), and expanded taxon set libraries based on the latest literature. MicrobiomeAnalyst 2.0 is available freely at microbiomeanalyst.ca. It contains comprehensive tutorials, equipped with a dedicated user forum (omicsforum.ca). The underlying MicrobiomeAnalystR package is also released (https://github.com/xia-lab/MicrobiomeAnalystR) to facilitate transparent and reproducible analysis.

## PROGRAM DESCRIPTION AND METHODS

**Figure 1.**
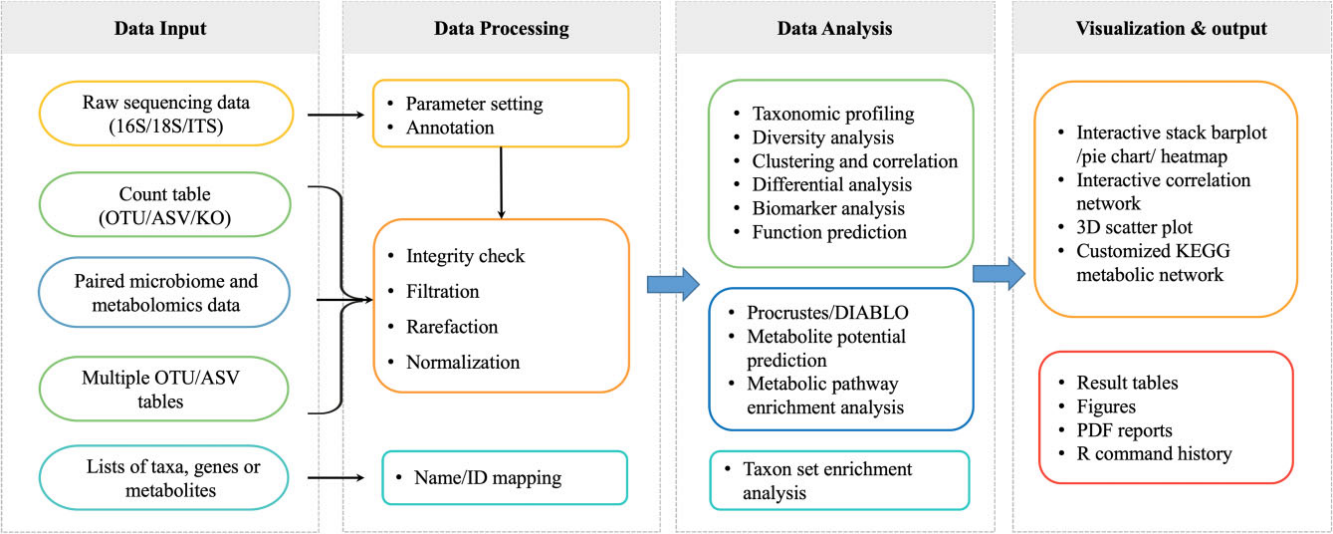
Workflow of MicrobiomeAnalyst 2.0.

The workflow of MicrobiomeAnalyst 2.0 consists of four main steps (Figure 1). It supports common input types including raw amplicon sequencing data for 16S, 18S rRNA genes or internal transcribed spacer (ITS) region, a single count table generated from maker gene or shotgun metagenomics, paired microbiome and metabolomic data tables or lists, multiple maker gene count tables from compatible studies, or taxonomic signatures. After upload, all input data follows the same general workflow of data processing, method selection, and result exploration. Comprehensive options and analysis support are available at each step. In the following sections, we will focus primarily on the new or improved features introduced in version 2.0.

### Amplicon sequencing data processing

High-throughput amplicon sequencing has yielded many insights into the development and progression of human diseases (3). It has become a ubiquitous method to study the complexity and diversity of microbiomes. Compared to shotgun metagenomics sequencing, the marker gene survey is both cost effective and computationally efficient, especially for highly heterogeneous communities with many low-abundant species. Raw reads need to be first processed into OTUs or ASVs before downstream analysis. Several tools have been developed for raw data processing including QIIME2 (22), Mothur (23) and DADA2 (24). However, command line skills are required to use these tools. MicrobiomeAnalyst 2.0 introduces a new module with an automated pipeline based on the well-established DADA2 workflow for processing amplicon sequencing data.

To start raw data processing, users can upload either single or paired-end compressed FASTQ files (.gz or .zip) from 16S/18S/ITS sequencing. A metadata file in plain text format (.txt or .csv) is also required for further downstream statistical analysis. The workflow includes filtering, dereplication, sample inference, chimera identification, and merging of paired-end reads. MicrobiomeAnalyst 2.0 provides a parameter selection page to allow users to tune processing parameters based on quality control graphical outputs. Taxonomy annotation is based on several reference databases, including SILVA (v138) (25), Greengenes (13.8) (26) and RDP (release 11.5) (27) databases for 16S sequencing, UNITE database (28) for ITS sequencing, and SILVA (v132) (25) for 18S sequencing. When raw spectral processing is complete, summary graphics and detailed processing information are generated for individual samples. The resulting ASV and taxonomy tables can be downloaded or directly used as input for marker data profiling by clicking the module redirection button.

### Integrative analysis for data from microbiome metabolomics studies

Metabolites are key players in microbial communications and interactions with their hosts. Metabolomics is increasingly used in recent microbiome studies to connect microbial community compositions and phenotypes at the level of altered metabolic processes (1,2,29). However, integrating high-dimensional microbiome and metabolomics data remains a major challenge. To address this gap, MicrobiomeAnalyst 2.0 introduces a new module to allow users to explore relationships between the microbiome profiles and their metabolic products.

Users can upload either paired abundance tables or paired lists. For microbiome data, the input features can be OTUs, ASVs or KEGG Orthologs (KOs). For metabolomics data, the input features can be metabolites (targeted metabolomics) or LC–MS peaks (untargeted metabolomics). For table inputs, different data filtering and normalization methods are provided based on the input data types. MaAsLin2 (19) and limma (30) are employed for the statistical comparisons of microbiome and metabolomics data, respectively. Both methods rely on general linear models to determine the associations between omics features and complex metadata, with support for covariate adjustments. List inputs are directly submitted to the name mapping step to prepare for the further integration analysis. Three strategies have been implemented for microbiome-metabolome integration—dimensionality reduction, metabolic network analysis, and correlation analysis.

#### Dimensionality reduction

Two robust dimensionality reduction methods, Procrustes analysis (PA) (31) and data integration analysis for biomarker discovery using latent components (DIABLO) (32), have been implemented to reveal overall patterns between paired microbiome and metabolomics datasets. PA is an unsupervised method that superimposes the principal components of two datasets by rotating the axes of one dataset until the maximum similarity is achieved. DIABLO is a supervised method that aims to identify multi-omics components that maximally explain the variances of individual data and their covariance together with the metadata of interest. The corresponding results are presented in an interactive 3D scatter plot. Users can switch between score plots, loading plots, and biplots to visualize high-level trends, highlight results with different metadata, or identify features of interest.

#### Metabolic network analysis

This module aims to offer metabolic analysis contextualized based on the taxa or KOs present in the uploaded microbiome profiles. Users can customize the global metabolic networks based on statistically significant taxa or all taxa detected in the microbiome data. Alternatively, users can choose the generic (unfiltered) metabolic background based on the aggregated microbial metabolic network, or its combination with the host metabolic network. Two well-established algorithms - mummichog (33) and globaltest (34) are used to perform enrichment analysis for LC-MS peaks and other features, respectively. The results are visualized in an interactive global metabolic network, in which nodes represent metabolites, edges represent enzymatic reactions, and reactions that fall outside of the study-specific microbial potential or KO profiles are greyed out. Users can click any enriched pathway names in the table to highlight the corresponding metabolites or KOs on the network. User can also directly click a node (metabolite) in the network to view the most associated microbes displayed as a circle plot.

#### Microbiome-metabolome correlation analysis

This module supports statistical, model-based and integrated correlation analyses. For statistical correlation analysis, the default option is the distance-based correlation method which can detect both linear and non-linear correlations (35). Other options include Pearson, Kendall, and Spearman correlations and their corresponding partial correlations. The results are summarized as an interactive heatmap. Pairwise correlation analysis often leads to a high number of false positives, making biological interpretation difficult. To address this issue, we implemented a model-based correlation based on >5000 high-quality genome-scale metabolic models (GEMs) to provide a probability heatmap between microbial taxa and their metabolites (36). Finally, users can choose to overlay the statistical and the model-based correlation heatmaps to integrate data-driven and knowledge-driven streams of evidence.

### Statistical meta-analysis across multiple microbiome studies

It is notoriously challenging to achieve reproducible features across different microbiome studies due to the variations in experimental design, analysis methods and quantitative assessment (37,38). The statistical meta-analysis module aims to provide a framework for integrating data from multiple maker gene studies of the same phenotypes to help identify robust and reproducible features.

The data upload and processing steps are similar to the single marker data profiling workflow, with an additional verification step to ensure that all datasets and metadata are consistent. After processing, batch correction is performed to adjust for potential technical variations to increase the comparability of different microbiome studies (13). After this step, three meta-analysis strategies are offered - visual exploration, biomarker meta-analysis, and diversity meta-analysis.

#### Visual exploration

This approach provides stacked area/bar plot and principal coordinate analysis (PCoA) plot to give an overview of high-level patterns, while still allowing users to investigate sample-level details. Stacked area/bar plot offers a sample-level profiling of taxa abundance across all datasets to better understand taxonomic composition, while PCoA provides an overview of the similarities/dissimilarities in microbial composition between samples and datasets. Please note that the previous ‘Projection to Public Data’ module has been migrated to this page.

#### Biomarker meta-analysis

The objective of this approach is to integrate the results from differential abundance testing of individual datasets to identify common microbial signatures associated with phenotype(s) of interest. The method is composed of two parts: abundance testing in individual datasets using multivariate linear regression followed by the integration of effect size using a random effects model based on the MMUPHin R package (13). The results are presented in the form of a bar plot displaying the top significant features along with a detailed table containing the statistical summaries of all features across individual studies.

#### Diversity meta-analysis

The approach integrates alpha and beta diversity indices across datasets. Common alpha diversity indices are computed for each study, and users can view ratios of indices between experimental groups using box plots and forest plots. Beta diversity indices are integrated by performing PCoA on common distance matrices from each study. Multiple statistical tests such as PERMANOVA (39), ANOSIM (40), PERMDISP (40) and MiRKAT (41) are available to measure significances on the effect of phenotype on community composition. Both graphical summaries and detailed tables are provided for alpha and beta diversity meta-analysis.

### Other features

#### Multi-factor analysis for complex metadata

Microbiome datasets continue to increase in size with more complex experimental designs, and therefore more complex metadata. In addition, complex metadata are especially important for observational studies, where both continuous and categorical covariates are often measured. Therefore, we have invested significant effort to enhance metadata support in MicrobiomeAnalyst 2.0. A metadata panel was implemented on the data integrity check page for users to inspect and edit metadata variables, including specifying whether they are continuous or categorical. Users can also specify the order of group labels for categorical metadata. A multi-factor comparison tool based on general linear models was implemented using the MaAsLin2 R package (19). Users specify their primary metadata of interest, and can include covariates such as age, sex or technical factors to adjust for. Covariates can be modelled as either fixed or random effects. A linear model containing the primary metadata and all covariates are fit to each feature, and then statistics are extracted from the model for the primary metadata.

#### Improved correlation analysis and function prediction

Several functions for marker gene profiling have been updated based on recent developments in the field. MicrobiomeAnalyst 2.0 now offers seven correlation methods for users to explore microbial relationships, including the recent Sparse Estimation of Correlations among Microbiomes (SECOM) method which provides measures of both linear and nonlinear relationships between microbes (10). For prediction of functional capacities from 16S rRNA gene abundance table, the previous version offered PICRUSt and Tax4Fun based on GreenGenes and SILVA taxonomy annotation, respectively. In version 2.0, we have updated the database for PICRUSt to support annotation of >200 000 OTUs against ∼7000 KOs. Tax4Fun2 is also available to allow users to predict potential functions directly from ASV sequences.

#### Enhanced visualizations for large data exploration

We implemented interactive plots for stacked bar/area plots and clustering heatmaps – those features are among the most frequent requests from our users for visual exploration of large datasets. Both mouse-over and zoom-in effects are supported to allow users to get details of the features/patterns of interest. Another improvement is the updated KEGG metabolic network (Release 105.0) for improved visualization and functional analysis.

#### Expanded taxon set libraries

The Taxon Set Enrichment Analysis (TSEA) module was created to allow researchers to identify taxonomic signatures characterized by their shared functions or associations with specific phenotypes to facilitate data interpretation and hypothesis generation. TSEA performs hypergeometric tests against a taxon set library of interest to detect the most frequently represented signatures from an input list of microbial features. In version 2.0, we have integrated data from popular databases such as gutMDisorder (42), GIMICA (43) and MiMeDB (44), and expanded the list of phenotypic features to include 102 microbiome features associated with immune responses, 77 microbiome-metabolite associations, 55 taxon sets associated with cancer, and 137 taxon sets associated with drug treatments. To improve the statistical power and biological relevance, we further consolidated taxon sets with at least four or more microbial members. The taxon set libraries now contain a total of 611 host-intrinsic features, 696 host-extrinsic features associated with diet, medication, and lifestyle, 500 associated with environmental features, and >700 single-nucleotide polymorphism (SNP) associated taxon sets.

### Case study

**Figure 2.**
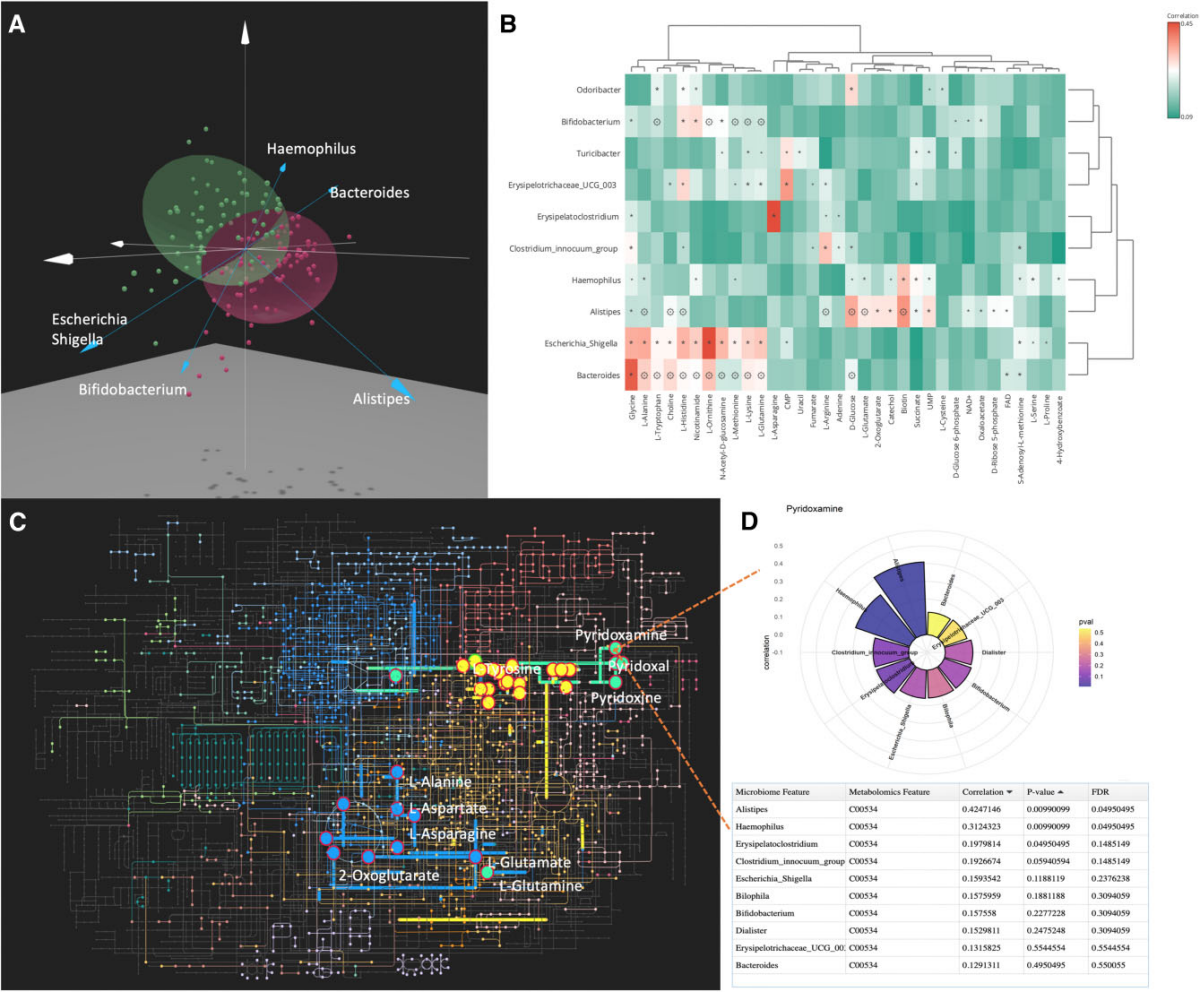
(**A**) DIABLO result visualized in 3D scatter plot. (**B**) Statistical correlation results overlayed with model-based correlation heatmap. Features passed the threshold of adjusted *P*-value 0.1 were used in this analysis. The color gradients indicate the statistical correlations and asterisks show the statistically significant correlation filtered by raw *P*-value 0.05. Diamonds indicate the correlations were also predicted by the GEM-based prediction models. (**C**) Pathway enrichment results based on KOs and metabolites (yellow: tyrosine metabolism; green: vitamin B6 metabolism; blue: alanine, aspartate and glutamate metabolism). (**D**) Circle plot with a detailed table below showing the most related taxa for the selected metabolite, *pyridoxamine*. The result was obtained by clicking the corresponding node.

To showcase the new features in MicrobiomeAnalyst 2.0, we leveraged a recent study on type 1 diabetes (T1D) (29). T1D is an autoimmune disorder that induces beta cell destruction and insulin deficiency (45). Previous studies showed that multiple factors can cause T1D such as genetic susceptibility, viral infections, dietary components, as well as gut microbiome (46). The objective of the study was to investigate the impact of altered microbial communities in people with and without T1D. Both 16S marker gene sequencing and LC–MS-based metabolomics were performed. Using the accession numbers provided in the original paper, we downloaded raw sequencing data from the NCBI Sequence Read Archive (SRA) database, and the metabolite concentration table from the MetaboLights (47). Raw data processing was performed using our DADA2 pipeline to get ASV abundance tables and taxonomy annotations. The result was submitted for functional profiling based on the prediction by Tax4Fun2 (7). Two types of co-analysis were then conducted by integrating metabolite abundance with either the ASV count data or the KO abundance table. The genus level was used as an example to explain the results presented in Figure 2.

Figure 2A shows the DIABLO biplot result presented in a 3D scatter plot. The composition of T1D and the control groups overlap to a certain degree which is consistent with the original publication. Several microbial taxa, such as *Bacteroides* and *Alistipes*, were observed to be associated with the top components. We hypothesize that these microbes drive the separation between T1D and non-diabetic subjects through certain metabolites. Detailed microbe-metabolite correlations are presented by the overlayed heatmap (Figure 2B). Only the features with an adjusted *P*-value <0.1 from the comparison analysis were used in this step. The statistical correlation was performed using the distance-based method and the AGORA database was selected for the GEM-based prediction result. With a significance cut-off of 0.05, we can observe that both approaches show *Bacteroides* significantly associated with glucose, glutamine, and several amino acids. *Alistipes* also correlated with a different set of amino acids, which is consistent with the pattern found by DIABLO analysis. Although *Bacteroides* was not identified as a biomarker in the original paper, however other studies have shown that it is related to diet and is a risk factor for early autoantibody development (48). Most studies focused on the compositional change of *Bacteroides* species in T1D without linking to function. Our analysis shows the metabolites significantly associated with *Bacteroides*, suggesting it potentially influences T1D through ‘Alanine, aspartate and glutamate metabolism’. Figure 2C shows the combined result of enrichment analysis from metabolites and KOs against the KEGG metabolic pathways using the globaltest method. Several pathways were detected by both datasets (highlighted in the left panel of Figure 2C) including ‘Vitamin B6 metabolism’, ‘Tyrosine metabolism’, and ‘Alanine, aspartate and glutamate metabolism’. The pathways that vary between the T1D and control group can be visualized within the network with different colors for each pathway. Taxa correlated with each metabolite can be visualized by clicking the corresponding node within the network. For example, the deficiency of pyridoxamine may impair insulin signaling (49). The top 10 most correlated genera such as *Alistipes* for pyridoxamine are shown in Figure 2D. We note that the metabolites within ‘Vitamin B6 metabolism’ were not significantly different between the T1D and the control groups, however the enrichment analysis can still identify the alteration at the pathway level.

### Implementation

The web interface of MicrobiomeAnalyst 2.0 is implemented based on the JavaServer Faces framework using the PrimeFaces library (https://www.primefaces.org, v12.0.0). The statistical functions and graphics are implemented using R (v4.2.2) and are freely available from the GitHub repositories (https://github.com/xia-lab/MicrobiomeAnalystR). To accommodate the growing user traffic and computing demand, the system is deployed on a Google Cloud instance load balanced with a second computing node hosted at McGill Data Center. For the raw data processing, the job submission and scheduling are based on the Simple Linux Utility for Resource Management (SLURM) system.

### Comparison with other tools

Several web-based tools have been developed for microbiome data analysis. Here we compare MicrobiomeAnalyst 2.0 with four other tools as well as to the previous version. Table 1 summarizes the main features of each tool. Popular tools dedicated to processing and archiving the raw sequence data, such as metagenomics rapid annotations using subsystems technology (MG-RAST) and MGnify (previously known as EBI Metagenomics) are not listed here (50,51). MicrobiomeAnalyst 1.0 (11) was developed to address the needs for statistical analysis by providing a comprehensive list of functions and publication-ready graphics. Similar tools include analysis of microbial population structures (VAMPS), Namco and MIAN (52–54). However, only the newly built Namco has a comparable number of analysis options as MicrobiomeAnalyst 2.0. Global catalogue of metagenomics (gcMeta) (55) is designed to annotate and analyze raw data from both marker gene and shotgun metagenomics, and is supported by a large collection of multi-omics studies, however it provides very limited analysis methods and no corresponding approaches for meta-analysis. Finally, integrative analysis of microbiome and metabolomics data has addressed an urgent demand by the microbial community. Overall, MicrobiomeAnalyst 2.0 is the most comprehensive web-based platform to allow user-friendly and streamlined microbiome data analysis and interpretation.

**Table 1.** Comparison of MicrobiomeAnalyst2.0 with other web tools. Symbols used for feature evaluations with ‘-’ for absent and ‘+’ for a more quantitative assessment (more ‘+’ indicating better support, e.g. better visualization and more options provided). The URLs for each tool are given below

Tools	MicrobiomeAnalyst		VAMPS	Namco	gcMeta	MIAN
	2.0	1.0				
Platform	Web	Web	Web (registration)	R Shiny	Web (registration)	Web (registration)
Input	FASTQ, count tables, BIOM, mothur	Same as 2.0 except FASTQ	FASTQ	FASTQ	FASTQ	Count tables; BIOM
Community profiling						
Diversity	+++	+++	+	++	–	++
Clustering	+++	++	–	++	–	–
Correlation	+++	++	–	++	–	++
Statistical analysis						
Processing	+++	+++	++	++	+	++
Comparison	Single/multi- factors	Single factor	Single factor	Single/multi-factor	Single factor	Single factor
Biomarker	++	++	–	++	+	+++
Time series	+	–	–	++	–	–
Functional profiling						
Functional prediction	PICRUSt, Tax4Fun, Tax4Fun2	PICRUSt, Tax4Fun	–	PICRUSt2	PICRUSt	–
Functional annotation	COG, KEGG, GEM	COG, KEGG	–	KEGG	KEGG	–
Network visualization	Contextualized	Generic	–	–	–	–
Co-analysis with metabolomics data						
Dimension reduction	Procrustes & DIABLO	–	–	–	–	–
Pathway enrichment	KO, targeted or untargeted metabolomics	–	–	–	–	–
Correlation analysis	Statistical and GEM based correlation	–	–	–	–	–
Meta-analysis						
Visual exploration	+	–	–	–	–	–
Statistical analysis	+	–	–	–	–	–
Integration with public data	+	+	+	–	–	–
Taxon set analysis	++	+	–	–	–	–

VAMPS: https://vamps2.mbl.edu/; gcMeta: https://gcmeta.wdcm.org; Namco: https://exbio.wzw.tum.de/namco; MIAN: https://miandata.org/projects.

### Conclusion

MicrobiomeAnalyst 2.0 has been developed to meet the fast-evolving needs of microbiome data analysis. It provides a web-based platform for researchers to easily explore and understand their data. To keep up with the latest developments, we have updated the libraries for functional annotation, taxon set enrichment analysis and embedded several recent statistical methods to enhance the modules developed in version 1.0. With the three new modules introduced in version 2.0, MicrobiomeAnalyst now supports streamlined analysis for marker gene data from raw data processing to downstream statistical and functional analysis. It also enables the integrative analysis for both paired microbiome-metabolomics datasets as well as multiple marker gene count tables. Our case study indicates that MicrobiomeAnalyst 2.0 can distill information from complex datasets to reveal the potential mechanic links between microbes and metabolites associated with T1D. Due to the internet bandwidth and large user traffic, the public server currently limits the maximum file size to 50MB for count tables and 100 raw sequence files per analysis session. We recommend using the MicrobiomeAnalystR package to researchers who plan to perform large-scale data analysis. In the future, we aim to support more type of analysis, such as single cell data analysis or casual inference within the context of host genetics (56–58).

## DATA AVAILABILITY

MicrobiomeAnalyst 2.0 is freely available without registration or login requirements at https://www.microbiomeanalyst.ca/.
